# An introduction to a special issue on the role of assistive technology in social inclusion of persons with disabilities in Africa: Outcome of the fifth African Network for Evidence-to-Action in Disability conference

**DOI:** 10.4102/ajod.v8i0.681

**Published:** 2019-11-12

**Authors:** Gubela Mji, Anthony Edusei

**Affiliations:** 1 Centre for Rehabilitation Studies, Department of Interdisciplinary Health Sciences, Faculty of Medicine and Health Sciences, Stellenbosch University, Cape Town, South Africa; 2 Kwame Nkrumah University of Science and Technology, Kumasi, Ghana

## Introduction and background

This article introduces the *African Journal of Disability* (AJOD)’s special issue on Disability and Inclusion in Africa: The Role of Assistive Technology. The special issue comprises papers presented at the fifth African Network for Evidence-to-Action in Disability (AfriNEAD) conference which focused on the role of Assistive Technology (AT) in social inclusion of persons with disabilities in Africa. The conference was held at Kwame Nkrumah University of Science and Technology (KNUST) in Kumasi, Ghana, in August 2017. The conference was a collaboration between AfriNEAD, based at the Centre for Rehabilitation Studies (CRS) at Stellenbosch University, as well as the Centre for Disability and Rehabilitation Studies (CEDRES) and the College of Health Sciences at KNUST. The KNUST College of Health Sciences, which holds a biannual conference, decided in 2017 to combine their efforts with those of AfriNEAD and CEDRES to table one unified conference. This joint venture gave an opportunity to raise awareness about disability-related issues to the College of Health Sciences. The intention was to facilitate and influence a response regarding disability issues at KNUST, at a national level in Ghana and beyond.

The theme for this combined conference was influenced by the fact that AT is being prioritised by the World Health Organization (WHO) through the Global Cooperation on Assistive Technology (GATE) project. Also, AfriNEAD is working on promoting contextually relevant research to inform policy and practice in this area. At the time of the conference, the Ghanaian government was focusing on the development of inclusive education policies to address the needs of persons with disabilities. Furthermore, the conference took place in Ghana when the country had demonstrated serious commitment regarding the domestication of the United Nations Convention on the Rights of Persons with Disabilities (UNCRPD) (UN 2006).

A conference focusing on AT was particularly relevant for the African continent as there is a general lack of research evidence regarding affordable, accessible, contextual and relevant AT, although AT is central to the well-being and livelihoods of people with disabilities. This lack of evidence undermines possible efforts that could assist the inclusion of many people with disabilities from participating fully in society, especially in Low- and Middle-income Countries (LMICs). Although many African countries have ratified the UNCRPD, it is not clear how governments of these countries plan to include the intention of the UNCRPD articles into policy and practice. The theme of this special issue of AJOD is appropriate for the advancement of knowledge of AT. The issue of AT is seen as a fundamental need for persons with disabilities in order to access other rights and needs, like health, education, employment, independent living and social participation. The special issue on AT, which is the first of its kind on the African continent, addresses the knowledge gap and will also stimulate further research and dialogue. It will also serve to publicise the important work conducted by AfriNEAD researchers and provide readers with more literature from an African context, as the papers are based on primary research conducted in different African countries.

## The African Network for Evidence-to-Action in Disability

The African Network for Evidence-to-Action in Disability is a flagship programme of the CRS at Stellenbosch University. Formed and inaugurated in November 2007, this regional research network was born out of the realisation that good disability research on its own cannot change the plight of people with disabilities in Africa but needs to embrace and combine strategic efforts of advocacy and activism, including the development of sustainable partnerships. It is the first network on the African continent that has focused on the issue of how disability research is translated into policy and practice for the realisation of the rights of persons with disabilities in Africa (Mji et al. 2009).

The African Network for Evidence-to-Action in Disability works towards the obliteration of the silo operation of different stakeholders by using research evidence as a tool in combining efforts of relevant sectors. From the standpoint of AfriNEAD, the challenge is clear, namely, translating research into evidence-based advocacy, policy, practice and products, particularly in the pan-African context, needs to be addressed systematically in a collaborative, co-ordinated, coherent and consistent manner (Mji et al. 2009). It is only when this happens that research evidence can act as a springboard for human rights instruments such as the UNCRPD. The African Network for Evidence-to-Action in Disability is increasing the participation of universities, Disabled Peoples Organisations (DPOS), business and civil society in the area of disability research within the pan-African region (Kachaje et al. 2014). At the core of the aims and objectives of AfriNEAD is the investigation into how disability research evidence influenced government. It also shows how that translated into policies, and then into practice to improve the lives of people with disabilities. The network points to one of its instruments for guidance, the Convention on the Rights of Persons with Disabilities (UNCRPD), which has now entered into international law and is perhaps the most significant – moral and practical – step towards realising the rights of persons with disabilities. The convention seeks to address discrimination, change perceptions and combat stereotypes and prejudices. Assistive devices feature strongly in the UNCRPD, with Articles 9, 19, 20, 25 and 26 giving clear indications regarding how to respond to the area of AT for people with disabilities.

The AfriNEAD conference is organised according to the 50 articles of the UNCRPD. After the 2007 conference, a team of researchers met to discuss the design and structure of the scientific component of the conference. Because AfriNEAD’ s focus was on the realisation of the rights of people with disabilities in Africa, and many African countries have ratified the UNCRPD, it stands to reason that the UNCRPD was the instrument of choice to guide researchers when preparing abstracts and papers for the conference. The 50 articles of the UNCRPD were combined to form eight areas of research, envisaging that presenters, through their papers, would generate evidence for these areas and thereby generate evidence for the UNCRPD. These areas are:

- children and youth with disabilities
- education: early to tertiary
- economic empowerment
- development processes in Africa: poverty, politics and indigenous knowledge systems
- health and HIV and AIDS
- systems of community-based rehabilitation
- wellness, sports, recreation, sexuality and spirituality
- research evidence and utilisation.

The outcome of presentations from these eight focus areas is synthesised into conference recommendations that are presented at a plenary session on the last day of the conference.

## Brief background of the current situation of assistive devices in sub-Saharan African countries

In sub-Saharan African countries and other LMICs, there are minimal to non-existent accurate statistical estimates on the availability of ATs for Persons with Disabilities (PWDs). Generally, in LMICs, the provision of assistive products is inadequate, with poorly structured systems in place to improve and facilitate service delivery (Borg, Larsson & Östergren 2011; Visagie et al. 2016b). Most often in such contexts, responses frequently exclude the intended beneficiaries, especially if indeed undertaken by consultants unfamiliar with the country in question (MacLachlan & Scherer 2018). Recent studies in Southern African countries have documented that only 15% – 25% of PWDs who need AT have access to it (Matter et al. 2016). Matter et al. (2016) further highlighted that studies carried out on ATs are not evenly distributed across the range of all impairments. The full range of ATs are often not available or evenly distributed to people who need them (Matter et al. 2016).

Both the challenges and limited successes that are reported provided a backdrop for the WHO GATE project of May 2016. One of the main aims of the GATE program was to increase access to high-quality and affordable assistive products or technology (WHO 2017). The GATE initiative describes its aim as to dramatically increase the historically appalling rates of access to AT, while also meeting the obligations of the UNCRPD and Sustainable Development Goals (SDGs).

In Africa, although there are many individual organisations advocating for ATs as part of their activities, these initiatives are often focussed regionally and/or on a specific impairment or disability. There is no joint African initiative which looks at comprehensive AT service delivery and all the related facets.

Systemic and institution-based bottlenecks related to the area of AT require a multifaceted approach such as that of AfriNEAD (MacLachlan et al. 2018). The inclusion of researchers, disability activists and advocates, as well as government and civil society, renders an inclusive forum to tackle some of these obstructions through constructive dialogue at tri-annual conferences (Mji et al. 2011). Some of the discussions in AfriNEAD conferences are related to some of the stigmas towards disability, which is a worldwide problem. Others put stress on culture and valuable innate indigenous resources that could be harnessed to promote AT systems. Bringing cultural resources such as the collective support of Ubuntu philosophy, with its strength of fostering harmonious relationships, has proven to be a supportive, empowering approach to AT services (Mji et al. 2011). In line with the WHO programme on AT, it is the right time for AfriNEAD and AJOD to publish research on AT from the African continent, both to inform audiences and to stimulate further research and practice in this area. The special issue provides policymakers from different sectors (education, health, social development and others) with consolidated evidence on the status of AT for persons with disabilities on the African continent.

## Major themes that emerged from the fifth African Network for Evidence-to-Action in Disability conference

The timing of the conference in relation to the attention given by WHO and the GATE programme to the area of AT, as well as the need for member countries to demonstrate to the UN how they have responded to UNCRPD, was opportune. The conference came as a special opportunity that brought together professionals from the medical, allied health, pharmacy, science, social science and engineering disciplines together with organisations of persons with disabilities, which significantly improved the perceptions of most of these professionals regarding disability issues.

The Centre for Disability and Rehabilitation Studies expressed appreciation of the opportunity to facilitate the hosting of the fifth AfriNEAD conference by KNUST. Not only did it offer an opportunity for the young graduates from the disability and rehabilitation studies programme to experience the feel of an international conference, but also to participate as presenters of scientific papers from their original research work. There were many benefits that Ghana, through KNUST and CEDRES, reaped from the conference (the conference was well-represented starting from the Office of the Asante King, as well as the Office of the President of Ghana and related ministers). One of the conference outcomes at KNUST was an advocacy group called ‘Advocates for Disability-Friendly KNUST’ – A for D-KNUST. Membership of the group comprises staff from CEDRES and other lecturers and administrators from KNUST. The group has created a WhatsApp platform to promote effective communication among its members, and has already elected its executives (AfriNEAD Conference Report 2018).

The fifth AfriNEAD conference generated 12 keynote addresses that came from researchers, people with disabilities, government representatives and non-governmental organisations. Sixty-eight papers were presented in the eight research areas. While many of the speakers were rehabilitation and medical professionals, their presentations were generally made from a stance that acknowledged that the rehabilitative potential of many interventions is limited by the lack of opportunities for disabled people to be included, and to be meaningful participants, in society. This mixture of papers concerned with ‘individual’, ‘medical’, ‘social’ and ‘emancipatory’ models was perhaps greater than at conferences outside Africa, where the lines of demarcation and engagement are more distinct, and perhaps less problematic. As mentioned earlier, AT, which was the focus of this conference, is seen as a fundamental need for persons with disabilities to be able to access other rights and needs like health, education, employment, independent living and social participation. Hence, abstracts that did not focus directly on research of AT, but rather on issues of inclusion of people with disabilities, were also accepted. For this editorial, the focus will be on the outcome that covers issues on AT. Table 1 lists key themes related to AT that emerged from the eight research areas.

**TABLE 1:** Key themes related to AT at the fifth African Network for Evidence-to-Action in Disability conference.

Specific themes related to the area of AT were the following: There is convincing evidence of the critical role of AT in enabling inclusive education; hence, the area of AT needs urgent attention. Governments should play a role in enabling universal access to essential and affordable assistive devices for young people with disabilities to be able to access and participate optimally in their education. There is a need for affordable assistive devices, for example, government subsidies, production of low-cost devices, devices to be readily available and accepted and appreciated by local people. Governments and other relevant sectors need to consider means by which persons with disabilities may be assisted to purchase and repair assistive devices at affordable costs. There is legislation to increase accessibility to AT, for example, *Assistive Technology Act* programmes in the United States. There is a need to address both the issues of the high cost of imported assistive devices and wheelchairs that come via donations, as they are not appropriate and not appreciated by the recipients. There is a need for strategies to improve communication and information dissemination between caregivers and professionals, and to link AT users with suppliers or providers. There is a need for a database or directory of AT suppliers and support networks, increasing general awareness of AT among a diverse range of stakeholders, building linkages between private and public sectors. There is a need to give attention to urine incontinence, catheter supplies and challenges of menstrual hygiene management among female adolescents with disabilities. Sign language needs to be introduced in nursing schools – and there is an urgent need for interpreters in the health sector. Access to AT for people with intellectual disabilities needs to be improved. There is a need for more research to determine the role of AT in disability inclusive development in Africa.

In summary, themes from the eight research areas focussed on the need for governments to play a role in enabling universal access to essential and affordable assistive devices for people with disabilities. There was a general concern about the high cost of imported assistive devices. It also became clear that more research is needed to determine the role of AT in disability-inclusive development in Africa.

## Papers reviewed for the special issue in the *African Journal of Disability*

Nine papers were reviewed for this special issue and only two papers finally completed the review process and were approved by the reviewers for publication. There were many reasons why some authors could not have their papers reach the point of being published, some of which were:

1. poor writing and research skills
2. high authorship fees
3. authors taking too long to respond to reviewers’ comments.

## A brief overview on the two papers that completed the review

The paper presented by Lyner-Cleophas focused on the value of AT for students pursuing studies and the role played by the Higher and Further Education Disability Services Association (HEDSA) in South Africa. The positive gains and existing gaps in disability inclusion in the higher education sector in South Africa are highlighted. The paper further highlights the important role of AT in fostering inclusion. The value of AT in education as facilitators for access to information cannot be underestimated as we strive towards social justice in South Africa and disability inclusion, particularly in the educational setting. The paper also emphasises the important role of networking across institutions to improve institutional knowledge and support to staff and students, with reference to ways in which barriers to learning can be overcome.

The second paper accepted for publication, written by Visagie et al., focuses on users’ perspectives on the AT-Info-MAP, a mobile application that maps AT sources in Africa. The WHO Disability Report states that around 15% of Africans are living with disability and experts estimate that the majority are in need of at least one assistive device. Lack of information about AT is one of the barriers that makes it difficult, if not impossible, to access AT. The AT-Info-Map aims to address this information gap with a mobile app that links AT suppliers with consumers in 10 countries in southern Africa. This 3-year project (2016–2019) is led by the Southern Africa Federation of the Disabled (SAFOD), in collaboration with Dimagi (technology partner), Stellenbosch University and the University of Washington.

## Conclusion

Although only two papers were accepted for publication after the rigorous process of peer review, they are very relevant and instrumental in providing information about the challenges and successes in the area of AT within the African continent. The two papers highlight gaps that research and practice on AT should focus on. On the other hand, the lack of scientifically sound papers and the capacity of authors to complete their papers for publication is a reason for concern in this area, especially given how critical AT is for participation and inclusion of people with disabilities in all spheres of life. There is a need for researchers to be supported with skills and resources to advance AT research in Africa. On the part of AfriNEAD, there is a need to ensure that presenters come to present at the conference while already working on the draft of their papers to be further developed for publication. There is also a need for the network to table either pre- or post-conference research capacity-building workshops to improve the research, writing and publication skills of AfriNEAD delegates, as many of them are novice researchers coming from under-resourced backgrounds.

## References

[CIT0001] AfriNEAD Secretariat, 2018, AfriNEAD Conference Report, Centre for Rehabilitation Studies, Stellenbosch University, Tygerberg.

[CIT0002] Borg J., Larsson L. & Östergren P., 2011, ‘The right to assistive technology: For whom, for what, and by whom?’, *Disability & Society* 26(2), 151–167. 10.1080/09687599.2011.543862

[CIT0003] MacLachlan M., Banes D., Bell D., Borg J., Donnelly B., Fembek M. et al., 2018, ‘Assistive technology policy: A position paper from the first Global Research, Innovation, and Education on Assistive Technology (GREAT) summit’, *Disability and Rehabilitation: Assistive Technology* 13(5), 454–466. 10.1080/17483107.2018.1468496 29790393

[CIT0004] MacLachlan M. & Scherer M., 2018, ‘Systems thinking for assistive technology: A commentary on the GREAT summit’, *Disability and Rehabilitation: Assistive Technology* 13(5), 492–496. 10.1080/17483107.2018.1472306 29772950

[CIT0005] Mji G., MacLachlan M., Melling-Williams N. & Gcaza S., 2009, ‘Realising the rights of disabled people in Africa: An introduction to the special issue’, *International Journal of Disability and Rehabilitation* 31(1), 1–6. 10.1080/09638280802280288 19194806

[CIT0006] Mji G., Gcaza S., Swartz L., MacLachlan M. & Hutton B., 2011, ‘An African way of networking around disability’, *Disability and Society* 26(3), 365–368. 10.1080/09687599.2011.560419

[CIT0007] Matter R., Harniss M., Oderud T., Borg J. & Eide A.H., 2016, ‘Assistive technology in resource-limited environments: A scoping review’, *Disability and Rehabilitation: Assistive Technology* 12(2), 105–111. 10.1080/17483107.2016.1188170 27443790

[CIT0008] Kachaje R., Dube K., MacLachlan M. & Mji G., 2014, ‘The African network for evidence-to-action on disability: A role player in the realisation of the UNCRPD in Africa’, *African Journal of Disability* 3(2), Art.#86, 5 pages. 10.4012/ajod.v3i2.86

[CIT0009] United Nations (UN), 2006, *Convention on the rights of persons with disabilities*, viewed 17 July 2018, from https://www.un.org/disabilities/convention/conventionfull.shtml. 10.1515/9783110208856.203 18348362

[CIT0010] Visagie S., Mlambo T., Van der Veen J., Nhunzvi C., Tigere D. & Scheffler E., 2016a, ‘Impact of structured wheelchair services on satisfaction and function of wheelchair users in Zimbabwe’, *African Journal of Disability* 5(1), a222 10.4102/ajod.v5i1.222 PMC5433454 28730049

[CIT0011] Visagie S., Eide A., Mannan H., Schneider M., Swartz L., Mji G. et al., 2016b, ‘A description of assistive technology sources, services and outcomes of use in a number of African settings’, *Disability and Rehabilitation: Assistive Technology* 12(1), 1–8. 10.1080/17483107.2016.1244293 27882821

[CIT0012] World Health Organization, 2017, *Global priority research agenda for improving access to high-quality affordable assistive technology*, World Health Organization, Geneva, Licence: CC BY-NC-SA 3.0 IGO.

